# Relative Impacts of Adult Movement, Larval Dispersal and Harvester Movement on the Effectiveness of Reserve Networks

**DOI:** 10.1371/journal.pone.0019960

**Published:** 2011-05-17

**Authors:** Arnaud Grüss, David M. Kaplan, Deborah R. Hart

**Affiliations:** 1 Institut de Recherche pour le Développement (IRD), UMR EME 212 (IRD/Ifremer/Université Montpellier 2), Centre de Recherche Halieutique Méditerranéenne et Tropicale, Sète, France; 2 Northeast Fisheries Science Center, Woods Hole, Massachusetts, United States of America; Institute of Marine Research, Norway

## Abstract

Movement of individuals is a critical factor determining the effectiveness of reserve networks. Marine reserves have historically been used for the management of species that are sedentary as adults, and, therefore, larval dispersal has been a major focus of marine-reserve research. The push to use marine reserves for managing pelagic and demersal species poses significant questions regarding their utility for highly-mobile species. Here, a simple conceptual metapopulation model is developed to provide a rigorous comparison of the functioning of reserve networks for populations with different admixtures of larval dispersal and adult movement in a home range. We find that adult movement produces significantly lower persistence than larval dispersal, all other factors being equal. Furthermore, redistribution of harvest effort previously in reserves to remaining fished areas (‘fishery squeeze’) and fishing along reserve borders (‘fishing-the-line’) considerably reduce persistence and harvests for populations mobile as adults, while they only marginally changes results for populations with dispersing larvae. Our results also indicate that adult home-range movement and larval dispersal are not simply additive processes, but rather that populations possessing both modes of movement have lower persistence than equivalent populations having the same amount of ‘total movement’ (sum of larval and adult movement spatial scales) in either larval dispersal or adult movement alone.

## Introduction

Spatial management of natural resources via the implementation of reserves has recently received significant attention in marine environments [1]–[3]. Movement of individuals among reserves and between reserves and surrounding unprotected areas is a major factor for determining population persistence in reserve networks [4]–[7]. Marine reserve implementation has historically concentrated on coastal environments, characterized by a larger proportion of populations with dispersing larvae and a relatively sedentary adult phase [8]–[10]. For this reason, considerable research effort has been directed towards the impact of larval dispersal on the functioning of marine reserve networks [4], [11]–[13]. However, the large scale implementation of marine reserve networks [14]–[17] and, in particular, the increasing interest in using reserves for populations that possess considerable adult mobility [18]–[20] have pushed questions of persistence for populations with different levels and forms of mobility to the forefront [21], [22]. The relative importance of larval dispersal versus adult movement for persistence and harvest of populations in the presence of reserve networks has not, to our knowledge, been rigorously examined in a comparative framework. In this paper, a simple conceptual metapopulation model is developed to compare the functioning of reserve networks for populations with different admixtures of larval dispersal and adult movement in a home range. Using populations that move exclusively in the larval phase, exclusively as adults or both, we develop analytic and numerical results to assess the relative impact of each on persistence and harvest, and to identify the driving forces underlying differences.

A number of modeling studies suggest that even relatively moderate adult spillover has a strong negative impact on reserve effectiveness in terms of persistence [23]–[26] and a positive impact on harvest under a relatively limited set of conditions [21], [27]–[29]. Moffitt *et al.* [25] develop a spatially-explicit model to examine persistence and harvest of a population that has dispersing larvae and adults moving within a home range. They find that adult movement has a significant impact on persistence in reserve networks, often for movement spatial scales significantly smaller than the reserve size. In particular, ‘network persistence’ (i.e., persistence due to the collective impact of a network of reserves as opposed to that due to any single reserve) is significantly and rapidly reduced by adult movement. Moffitt *et al.* [25] also suggest that larval spillover has greater potential to improve harvest than adult spillover. Le Quesne and Codling [29] find the opposite using a model including harvester movement in response to prey density, but only the special cases of non-dispersing larvae and a uniform spatial distribution of larvae are considered.

While these results indicate the importance of adult movement for population dynamics in reserve networks, the underlying mechanisms driving differences in the effects of larval and adult connectivity and the generality of these effects have not been clearly identified. In this paper, we build on the approach of Moffitt *et al.* [25] by including a number of key modifications that provide a rigorous general conceptualization of the impacts of these different forms of connectivity on the conservation and harvest effects of marine reserves. A single functional form is used for both larval dispersal and adult movement, providing a comparative platform for evaluating which process has a greater impact on persistence and harvest. Analytic results identify the underlying mechanism behind differences between the two, as well as the universality of this mechanism. Furthermore, we examine in detail consequences of the movement of harvesters to take advantage of spillover and the redistribution of harvest effort previously in reserves to remaining non-reserves areas, both of which have been widely recognized as important for population dynamics and harvest in reserve networks [13], [30]–[32]. In particular, harvester behavior potentially interacts differently with adult movement and larval dispersal because individuals that have spilled over are exposed to harvest at different points in their life history. Our results indicate that harvester movement changes not only quantitatively, but also qualitatively, differences in the impact of reserves on populations moving as adults versus as larvae.

## Methods

We begin the development of our spatial metapopulation model by first considering a simple *non-spatial* population where each individual produces on average a certain number of eggs, *b(f)*. Individual egg production is a function of life-history parameters and the instantaneous harvest rate, *f*. These eggs become larvae that experience intra-cohort, density-dependent interactions before entering the adult population. This population structure is represented by:
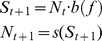
(1)where *N_t_* is the number of adult individuals at time *t*, *S_t_* is the number of pre-recruits (i.e., fish individuals that are prepared to recruit into the adult population, but have not yet done so; also referred to as ‘settlers’), and the function 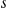 represents intra-cohort density-dependent processes that connect the number of pre-recruitswith the final number of adult individuals. While directly applicable to semelparous populations that reproduce once before dying, this population structure is commonly used in fisheries to represent age-structured populations at equilibrium [12]. In this latter case, *b(f)* represents the average egg production of a recruit over its lifetime, here referred to as the per recruit egg production, *N* represent the number of new recruits to the population, and *t* is a generational time step, as opposed to a physical unit of time.

This population structure is adapted to spatially-distributed, sedentary populations with dispersing larvae through the introduction of a dispersal matrix [12]:
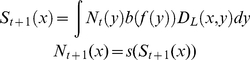
(2)where the dispersal function, *D_L_(x,y)*, expresses the probability that larvae produced by adults at one location, *y*, will eventually settle in another location, *x*. Intra-cohort density-dependent mortality, represented by the function *s*, is applied to pre-recruits after they have arrived in their future adult habitat and, therefore, is only a function of the local number of settling larvae *S*. The function *s* does not explicitly depend on location, implying that settlement habitat is assumed of uniform quality over space. Note that the harvest rate, *f*, varies as a function of location due to the presence or absence of reserves.

In order to integrate the movement of adults in a home range in this model, we must first differentiate between two concepts of the harvest rate. The first is ‘harvest rate’, *f(x)*, the rate of removals at location *x*, which depends on the distribution of harvesters. The second is the harvest rate experienced by fish individuals as a function of the center of their home range. If individuals move in a home range, then they may be caught away from the center of their home range, and therefore the biological consequences of this harvest will be felt elsewhere than the actual location of capture. This ‘effective’ harvest rate [24], [25], *f_eff_(x)*, of individuals whose home range is *centered* at a location, x, is given by:
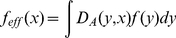
(3)where *D_A_(y,x)* represents the probability that an individual whose home range is centered at *x* is found at a given moment at location *y*. This ‘effective harvest rate’ determines the biological dynamics of the system and is integrated into the model by replacing *f* with *f_eff_* in Equation (2):
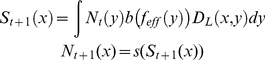
(4)Equation (4) implicitly assumes that adult individuals produce their eggs at the center of their home range, as is the case for breeding sea birds and many terrestrial animals, but likely not the case for many mobile marine species (e.g., live-bearing sharks). Larval dispersal via the movement of adults would be included in the model in an identical fashion to other forms of larval dispersal (Equation (2)), and, therefore, is not separately addressed here. Nevertheless, this possibility is implicitly addressed by examining populations with different mixes of both larval dispersal and home-range movement.

### Harvest and the spatial (re)distribution of harvest effort

As with harvest rate, we can distinguish between two measures of the harvest at a location, one as perceived by harvesters, the other as perceived by biological populations. We assume that each recruit contributes on average a certain harvestable biomass over its lifetime, here referred to as the harvest-per-recruit *h* (also known as yield-per-recruit in the fisheries literature). The total harvest of the system is the product of harvest-per-recruit and the number of recruits to the system:
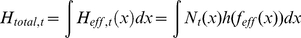
(5)where 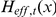, the effective harvest at a location, represents the biomass caught whose home-range center is at *x*. The actual biomass caught by harvesters at a given location, *H_t_(x)*, is obtained from the effective harvest by inverting the adult home-range distribution:
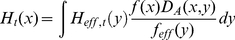
(6)


We consider two different scenarios for the spatial distribution of effort in the presence of reserves. For both scenarios, harvest effort, which is assumed proportional to the harvest mortality rate, *f(x)*, is zero inside reserves. Outside reserves, effort can either be uniform (i.e., *f(x) = f* for all *x* not in a reserve), or the effort distribution can change in response to the expected harvests at a location. This latter effect is modeled using a gravity model [26]:
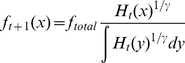
(7)where *f_total_* is the total harvest mortality integrated over all locations and 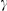 is a measure of the difference among harvesters in perception of benefits of operating at a location. Small values of 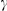 produce effort that is highly concentrated in areas of increased harvests.

We also consider two different scenarios for the fate of effort that was in reserves before they were closed. Either this effort ‘disappears’ or it is fully redistributed to the remaining non-protected areas at the time of reserve creation (the ‘fishery squeeze’ assumption, [13], [31]). Combining these two scenarios of harvest redistribution after reserve implementation with the two scenarios for the spatial distribution of harvest effort produces a total of four scenarios for the response of effort to reserve implementation, ranging from uniform effort distribution that diminishes after reserve creation in proportion to the amount of area in reserves, to total harvest effort that is conserved before and after reserve creation and effort that changes spatially in response to expected harvests. The last scenario evoked is the most likely to occur in the real world except in cases of extremely low mobility fisheries and/or simultaneous changes in conventional harvest management to reduce total harvest effort. However, uniform effort distribution and effort disappearance after reserve creation have generally been the norm in marine reserve modeling studies until relatively recently [21]. Furthermore, consideration of these two scenarios allows us to analyze the relative impacts of harvester movement on the effectiveness of reserve networks and to highlight the erroneous conclusions that could be made if ‘fishery squeeze’ and/or harvester behavior are ignored when they actually occur.

### Model application

In order to gauge the sensitivity of model results to life-history traits of the populations modeled, we apply our spatial metapopulation model to three different life-history configurations, each of which is roughly modeled on a real population. It is important to emphasize that for each population only growth, reproduction and natural mortality parameters are modeled after the corresponding real population. Both larval dispersal and adult home-range movement are considered for each irrespective of the type and nature of connectivity in the real populations.

The three populations that serve as the basis for our model simulations are: U.S. canary rockfish (*Sebastes pinniger*) and skipjack (*Katsuwonus pelamis*) and yellowfin tuna (*Thunnus albacares*) populations of the Atlantic Ocean. U.S. canary rockfish is a long-lived, iteroparous fish population whose first reproduction occurs at approximately eight years old, 3 years after initial vulnerability to harvest [33], making the population particularly susceptible to overexploitation [34] and a target for management with reserves. Rockfish are often territorial and their movements are generally well represented by a home range [35], [36]. Skipjack and yellowfin tunas of the Atlantic Ocean are relatively short-lived, iteroparous fish populations whose reproduction occurs, respectively, before and after age of first harvest [37]. Tuna movements are far more complex than a simple home range, including significant migratory behavior [38], though there is some precedent for representing their large-scale movements as diffusive [39], [40] and some argue that over long time scales diffusive movements can be approximated as a home range [21], [22]. Here we make *absolutely* no claim to be representing tuna movement. Rather we are using non-movement life-history parameters of these species so as to have three significantly different patterns of growth, mortality and reproduction to test sensitivity of model results to these *non-movement* parameters. So as to make clear that we are not attempting to model all aspects of the life-history of these species, we hereafter refer to canary rockfish, skipjack tuna and yellowfin tuna as the ‘long-lived’, ‘harvest-first’ and ‘spawn-first’ species, respectively.

**Figure 1:**
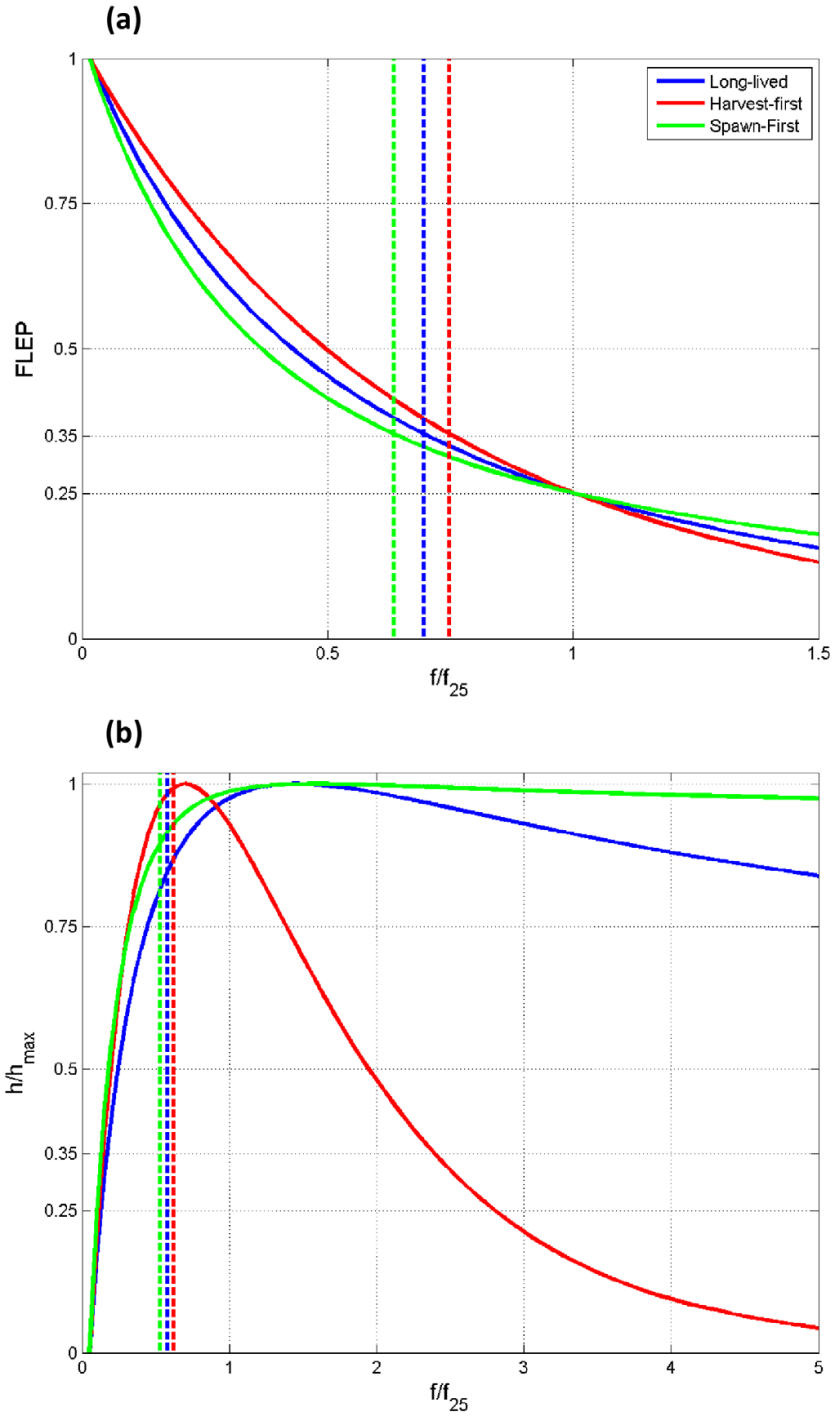
Fraction of lifetime egg production (FLEP, i.e., per recruit egg production/natural per recruit egg production) ((a)) and harvest-per-recruit over maximum harvest-per-recruit (*h*/*h_max_*) in function of harvest mortality over fishing mortality ((b)) when lifetime egg production is at 25% of its unfished value (*f*/*f_25_*) for the three species studied in the present study. The dashed lines represent the harvest mortality above which the studied species collapse in the absence of reserves (i.e., the harvest mortality for which lifetime egg production is at 35% of its unfished value in the context of this paper).

For the long-lived species, individuals are assumed here to recruit to the population at age 0 and to grow according to a von Bertalanffy growth function:
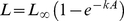
(8)where *A* is the age of the individual, *k* is the Brody growth coefficient, and *L_∞_* is the maximum length. For the other two species, empirical relationships from the literature are used to relate length to age (Appendix S1, [41], [42]). For all three populations, biomass and reproductive capacity at a given age are assumed to be allometic functions of length (i.e., each is proportional to *L^n^* for some exponent *n*). See Table 1 for a list of population parameter values, and Figures 1 and prior literature [12], [43] for the per recruit egg production and harvest-per-recruit as a function of harvest rate.

**Table 1:** Non-movement parameter estimates for the long-lived (canary rockfish - *Sebastes pinniger*), harvest-first (yellowfin tuna - *Thunnus albacares*) and spawn-first (skipjack tuna - *Katsuwonus pelamis*) species.

Parameter	Definition	Estimate	References
***Long-lived species***			
α	Allometric biomass parameter	3.03	[63], [64] (estimate of α for a related species, *Sebastes alutus*)
β	Allometric reproductive-capacity parameter	4.1416	[33]
L_∞_ (cm)	Maximum length	53.4	[33]
k (year^−1^)	Brody growth coefficient	0.183	[33]
m (year^−1^)	Natural mortality rate	0.06	[33]
A_F_ (years)	Age of first harvest	5	[33]
A_50_ (years)	Age of first reproduction	8	[33]
***Harvest-first species***			
α	Allometric biomass parameter	2.976	[65]
β	Allometric reproductive-capacity parameter	2.9861	[66] (estimate of β for the yellowfin tuna population of the Indian Ocean)
m (year^−1^)	Natural mortality rate	0.6	[67]
A_F_ (years)	Age of first harvest	0.28	[68]
A_50_ (years)	Age of first reproduction	2.63	[37]
***Spawn-first species***			
α	Allometric biomass parameter	3.253	[69]
β	Allometric reproductive-capacity parameter	2.5704	[70]
m (year^−1^)	Natural mortality rate	0.8	[71]
A_F_ (years)	Age of first harvest	2.13	[68]
A_50_ (years)	Age of first reproduction	2.08	[37]

Harvest mortality is gauged in this paper in terms of its effect on per recruit egg production. For all three species, a pre-reserve harvest mortality rate that reduces per recruit egg production to 25% of the unfished value is used. This value represents a heavily exploited species and is consistent with levels for several California rockfish species [44] A hockey-stick density-dependent recruitment relationship [45] is parameterized so that in the absence of reserves the population collapses (i.e., population size becomes too small to support a fishery) when harvest mortality reduces the per recruit egg production below a certain value (hereafter referred to as the ‘critical per recruit egg production’) [46]. The value of this collapse point may range between 10 and 60% depending on the species [47]. A value of 35% is consistent with those found for several rockfish species [47] and will be used here when not explicitly varying this parameter. Given this collapse point, harvests are not sustainable in the absence of reserves. Qualitative aspects of our results are generic and not tied to the particular settler-recruit relationship or collapse point used. So as to be able to oppose the effects of adult movement to those of larval dispersal, we assume that the larval dispersal kernel and the adult home-range have identical functional forms. Reserves occur periodically along an infinite, one-dimensional space, and dispersal and home-range functions are given by a Laplacian distribution:
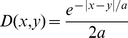
(9)where *a* is the mean movement distance.

## Results

**Figure 2:**
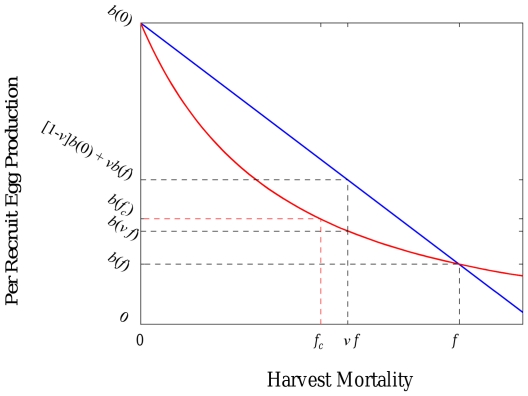
Per recruit egg production as a function of harvest mortality rate for the long-lived species (red curve). Immediately after reserve implementation, changing the fraction of habitat in reserves moves the average reproductive capacity on the blue line for a population with dispersing larvae and sedentary adults. For a population with adults moving within a home range and non-dispersing larvae, changing the fraction in reserves moves the reproductive capacity on the red curve. Consequently, when lifetime egg production is a decreasing, convex function of harvest mortality, adult movement leads to lower egg production immediately after reserve implementation than larval dispersal. Per recruit egg production functions are, respectively, more and less convex for the harvest-first and spawn-first species, but similar qualitative results are obtained for these species.

Before proceeding to numerical evaluation of the model, we begin with some general analytic results that provide insights into how larval dispersal and adult movement affect persistence. Consider first the system immediately after reserve creation, so that adult density and harvest effort are still uniform over space. For populations with only larval dispersal, the number of settlers arriving at a given location at the next time step is:
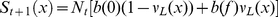
(10)where *f* is the harvest rate outside protected areas after reserve implementation and 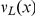 is the fraction of larvae arriving at *x* from fished areas 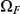:
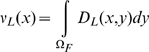
(11)If we now consider the same system with only adult home-range movement, the number of settlers becomes:
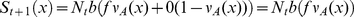
(12)where 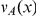 is the fraction of time an individual centered at x spends in fished areas 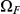:
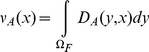
(13)Assuming that the larval dispersal and adult movement distributions are the same, symmetric around *x* and uniform over space (as is the case for the Laplacian distribution in Equation (9)), Equations (10) and (12) are similar except that larval dispersal linearly mixes egg production inside and outside reserves, whereas adult home-range movement linearly mixes the harvest rate inside and outside reserves. As the relationship between harvest rate and per recruit egg production is decreasing and convex (see proof in Appendix S2), the number of eggs produced in the adult movement case will necessarily be lower than in the larval dispersal case by Jensen's inequality (Figure 2), suggesting that final equilibrium persistence will also be lower for adult movement.

Next consider the limiting cases of large dispersal distance or home-range size (or equivalently, very small reserves). In this limit, 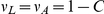, where *C* is the fraction of habitat in reserves, and settlement is uniform over space so that global persistence is guaranteed if the number of settlers in Equations (10) and (12) exceeds the fraction of natural settlement necessary to avoid collapse (e.g., 35%). As we have just shown that the number of eggs produced will be greater for larval dispersal than adult movement, persistence of these populations will occur at lower closure fractions for the larval dispersal case than for the adult movement case. For larval dispersal, persistence occurs if [12]:
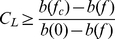
(14)where *f_c_* is the harvest rate that reduces per recruit egg production to the critical level in the absence of reserves. For adult movement one finds:
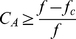
(15)The same fraction of habitat in reserves is required for the two cases only if reproductive capacity is a linear function of harvest rate (e.g., 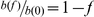). For more realistic scenarios (i.e., decreasing, convex functions), more habitat is required in reserves for adult movement than for larval dispersal. For example, for the long-lived species with 25% natural per recruit egg production remaining in fished areas, persistence for large dispersal distances occurs if greater than 13% of habitat is in reserves, whereas for large home ranges persistence requires at least 31% in reserves.

**Figure 3:**
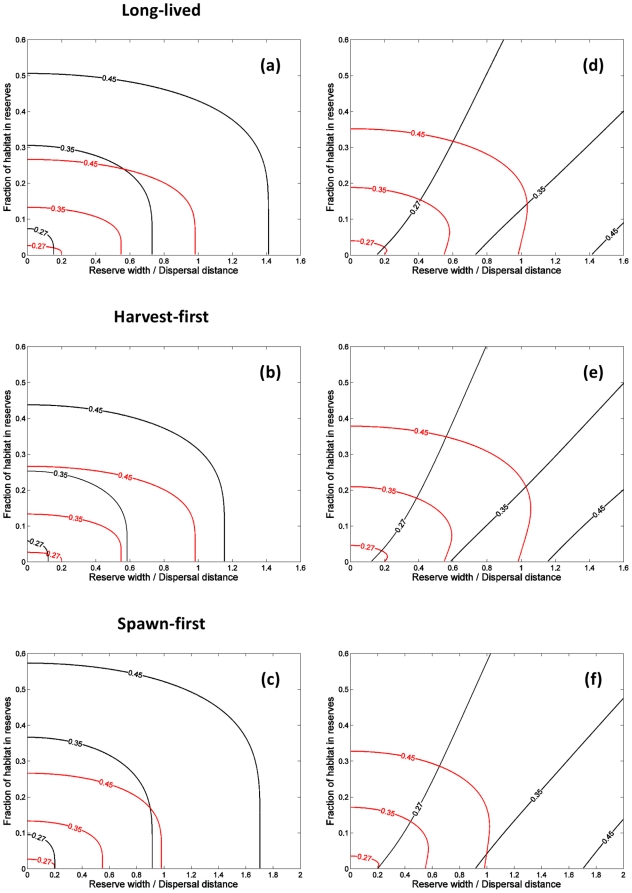
Border between persistence and collapse in the adult movement case (black curves) versus the larval dispersal case (red curves) as a function of reserve width (in units of the dispersal distance or home-range size) and fraction of habitat in reserves. In all cases, collapse occurs for very small reserves covering a small fraction of habitat (lower, left corner of panels). Harvest effort is uniformly distributed outside reserves. (**a,d**) are for the long-lived species, (**b,e**) for the harvest-first species and (**c,f**) for the spawn-first species. For (**a,b,c**), it is assumed that the effort that had previously been in the reserves disappears at the time of reserve creation, while for (**d,e,f**) it is assumed that the total effort does not change before and after reserve creation. Per recruit egg production is 25% of its unfished value in harvested areas, and three different values of the critical per recruit egg production below which collapse occurs in the absence of reserves are shown (27, 35 and 45% of the natural per recruit egg production).

Consider these results for the case when harvest effort redistributes uniformly in non-protected areas after reserve implementation (i.e., ‘fishery squeeze’ occurs). In such a system the harvest mortality rate in non-protected areas is:
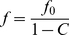
(16)where *f_0_* is the pre-reserve harvest rate [13], [27]. Replacing *f* by 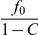 in Equation (14) logically yields that persistence in the larval dispersal case requires more habitat area in reserves when fishery squeeze is considered. However, even with effort redistribution, there is always a value of 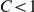 for which persistence occurs. For example, for the long-lived species, persistence for large larval dispersal distances occurs if at least 19% of habitat is in reserves versus 13% when fishery squeeze is ignored. Replacing *f* by 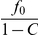 in Equation (15), it follows after simplification that for large home-range sizes, persistence requires that:
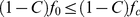
(17)Since 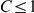, persistence is ensured if and only if 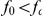. Hence, no fraction of habitat in reserves (<1) will cause persistence if the pre-reserve harvest rate is greater than the collapse point (vertical axes of Figures 3d–f).

Analytic results can be found for persistence for arbitrary reserve widths and fractions in reserves for both the larval dispersal [12], [48] and adult movement cases. Whereas in the larval dispersal case, persistence is a complex function of the connectivity between reserve and non-reserve areas [48], in the adult movement case, subpopulations are not connected through dispersal and, therefore, global persistence is guaranteed whenever there is at least one location where 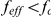. As reserve centers are the locations of the system where persistence is most likely, whether the population of interest will ultimately be persistent can be determined by evaluating if 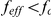 at reserve centers (Appendix S3). For all species life-histories examined, persistence requires considerably larger total fraction in reserves and/or larger individual reserves for a given home range than for an equivalent larval dispersal distance (Figures 3a–c), particularly in the limit of large dispersal distances or home-ranges discussed above (along vertical axes in Figures. 3). Perhaps most importantly, if fishery squeeze occurs (Figures 3d–f), patterns of persistence are qualitatively different for the larval dispersal case than the adult home-range case, with the latter requiring large reserve widths and, paradoxically, small fractions of habitat in reserves.

**Figure 4:**
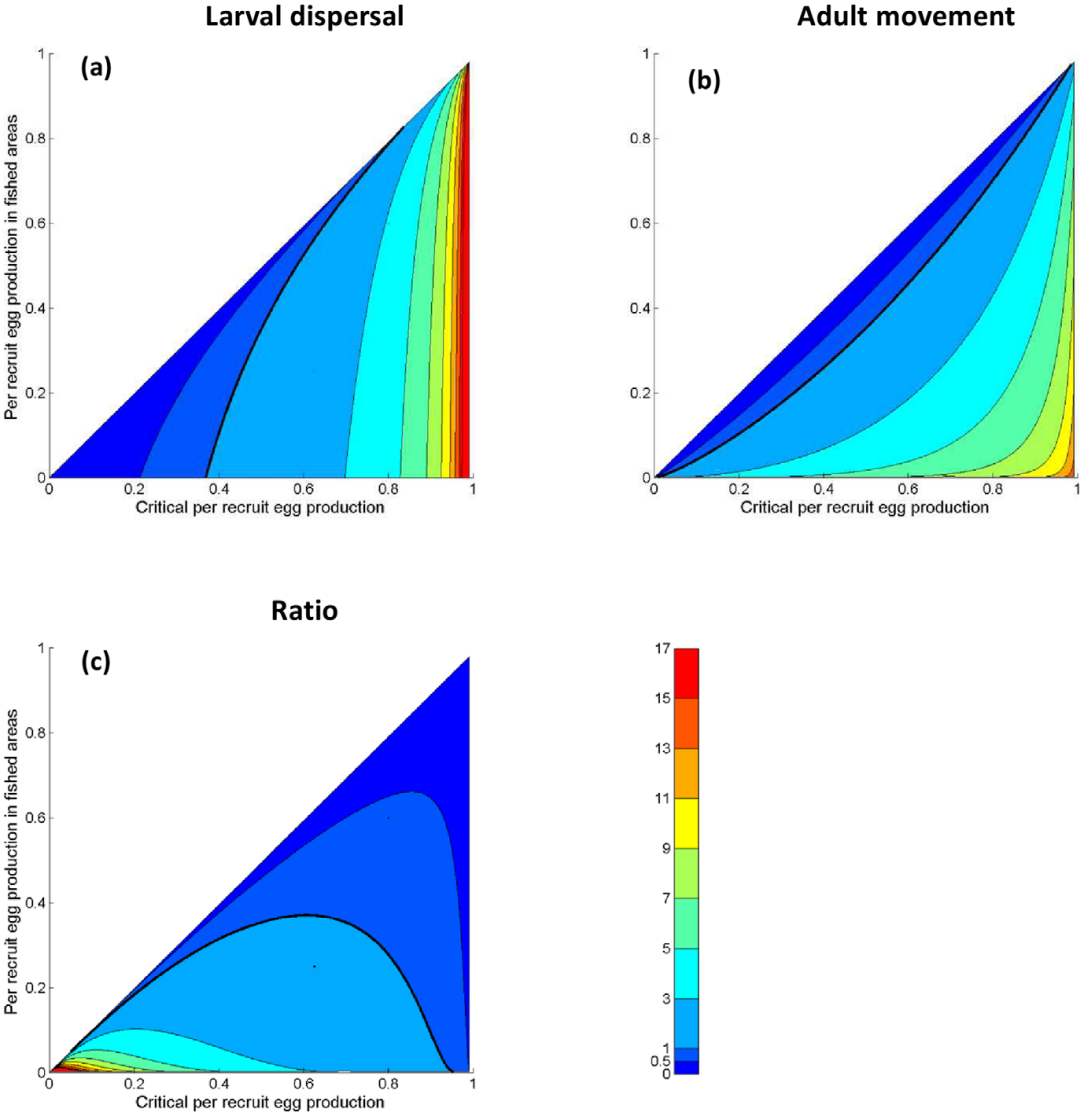
Minimum reserve width (in units of the dispersal distance or home-range size) required for persistence of an isolated reserve as a function of critical per recruit egg production and per recruit egg production in harvested areas for the long-lived species. (**a**) is for larval dispersal alone, (**b**) is for adult movement alone, and (**c**) gives the ratio of these two quantities, with values greater than one indicating larger reserves are needed to ensure persistence for the larval dispersal case than for adult movement. Here harvest effort is assumed uniformly distributed outside reserves and the effort that had previously been in the reserves disappears at the time of reserve creation. Note that similar qualitative results are obtained for the harvest-first and spawn-first species.

In the limit of a single isolated reserve (along the horizontal axes in Figures 3), differences are also significant, except for when the value of per recruit egg production in fished areas is close to the critical per recruit egg production (e.g., 25 and 27% of the unfished per recruit egg production, respectively). Examining in more detail the limit of a single isolated reserve (Appendix S3 and Figures 4), one finds that minimum reserve widths for persistence are generally smaller for larval dispersal than adult movement for realistic values of the critical per recruit egg production (i.e., 0.1–0.6), but can be larger for high critical values and/or per recruit egg production in harvested areas close to the critical value (Figure 4c).

Patterns of persistence in reserve networks are qualitatively similar for the three species studied. There are somewhat more reserve configurations leading to persistence for the harvest-first species than for the two other species (Figures 3b and e), and slightly fewer for the spawn-first species than for the two other species (Figures 3c and f). These quantitative differences are tied to the functional dependence of reproductive capacity of each species on harvest mortality rate (Figures 1a and 2).

### Persistence and harvest for different scenarios of harvester movement

**Figure 5:**
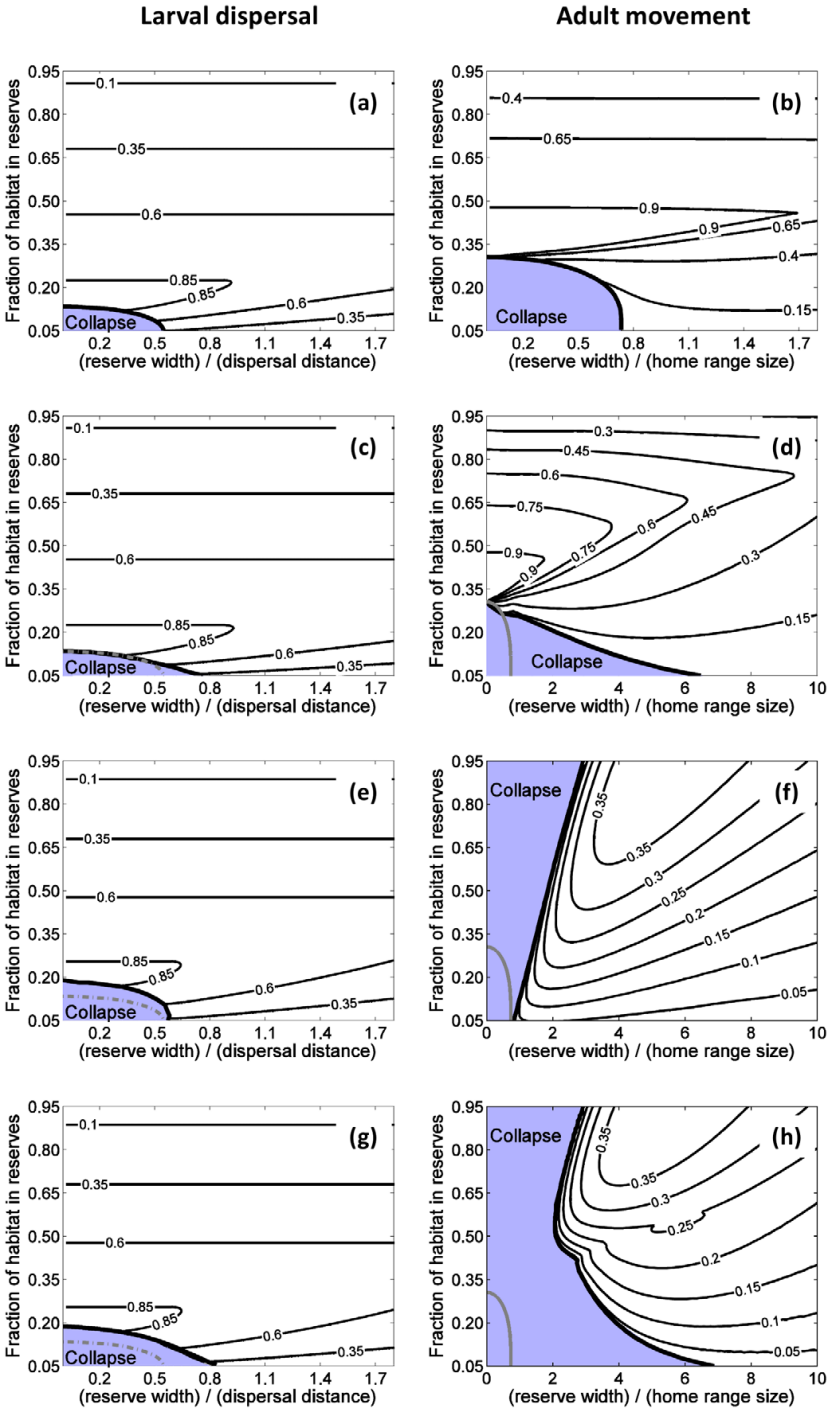
Equilibrium harvest as a function of reserve width (in units of the dispersal distance or home-range size) and fraction of habitat in reserves for the long-lived species. Panels to the left are for populations with sedentary adults and dispersing larvae, while panels to the right are for populations with mobile adults and non-dispersing larvae. For (**a,b,c,d**), it is assumed that the effort that had previously been in the reserves disappears at the time of reserve creation, while for (**e,f,g,h**) it is assumed that the total effort does not change before and after reserve creation. For (**a,b,e,f**), harvest effort distribution is uniform outside reserves, while for (**c,d,g,h**), it depends on local expected harvests and the value of γ is 1.2. The light blue area represents reserve configurations leading to a collapsed population and for (**b,c,d,e,f,g,h**) the dash-dotted grey line represents the border between persistence and collapse when harvester behavior and effort redistribution after reserve creation are both ignored. Harvest values shown are relative to the maximum value for the adult movement case when harvester behavior and effort redistribution after reserve creation are ignored.

**Figure 6:**
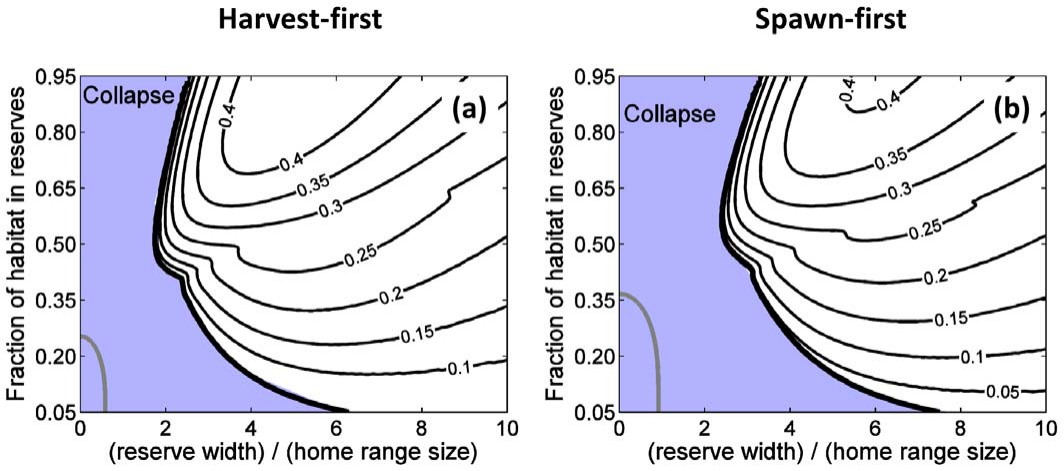
Equilibrium harvest as a function of reserve width (in units of home-range size) and fraction of habitat in reserves for populations with mobile adults and non-dispersing larvae. (**a**) is for the harvest-first species and (**b**) for the spawn-first species. Here it is assumed that the total effort does not change before and after reserve creation and that harvest effort distribution depends on local expected harvests, and the value of γ is 1.2. The light blue area represents reserve configurations leading to a collapsed population the grey line represents the border between persistence and collapse when harvester behavior and effort redistribution after reserve creation are both ignored. Harvest values shown are relative to the maximum value for the adult movement case when harvester behavior and effort redistribution after reserve creation are ignored.

Numerical model evaluation is required to examine patterns of persistence when harvest effort is non uniform outside reserves and to obtain total harvest levels. Given the qualitative similarities in patterns of persistence (Figures 3) between the three species studied, we focus on the results for the long-lived species (Figures 5), for which patterns of persistence appear to be intermediate between those of the two other species. Results for the other species showed only relatively minor quantitative differences (Figures 6).

**Figure 7:**
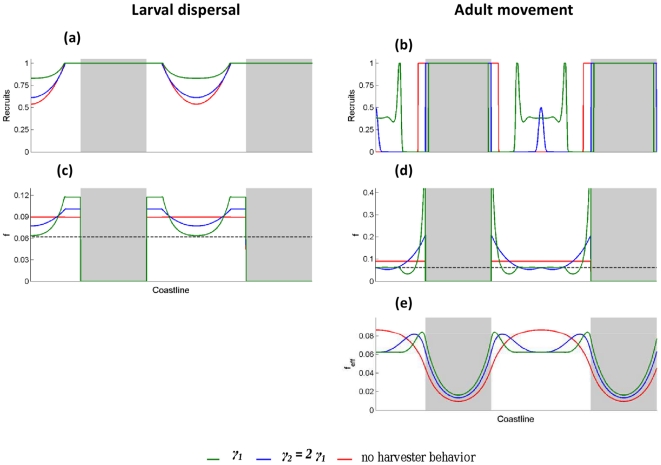
Spatial patterns of (a,b) recruitment, (c,d) real harvest mortality rate (*f*), and (e) effective harvest mortality rate (*f_eff_*) for a system of periodically-spaced, uniformly-sized reserves (grey areas) at equilibrium for the long-lived species. (**a,c**) are for populations possessing only larval dispersal, whereas (**b,d,e**) are for populations that only have adult movement in a home range. The effective mortality rate is not shown for the larval dispersal case as it is identical to the real harvest mortality rate. Harvest effort is uniform outside reserves for red curves. For the green and blue curves, the harvest effort distribution in the non-protected areas depends on local expected harvests, with the value of γ being 1.2 for green curves and 2.4 for blue curves. The units of recruitment are arbitrary, but consistent between simulations. The dashed black line on (**c**) and (**d**) represents the harvest mortality rate above which the population collapses in the absence of reserves.

Non-uniform harvest effort in response to expected harvests at each location reduces the set of reserve network configurations that produce persistent populations for both the case of exclusive larval dispersal and that of exclusive adult movement in a home range (Figures 5c–d). Nevertheless, the reductions in persistence are considerably more drastic for adult movement. For the larval dispersal case, spatial heterogeneity in recruitment is capped by the density-dependent settler-recruit relationship (Figure 7a). This limits the extent of effort concentration in areas along reserve borders (Figure 7c) and therefore only marginally changes persistence. For the adult movement case, there is no cap in our model on the number of individuals using a particular location as part of their home range. Furthermore, harvests along reserve edges are driven by the spillover of individuals from reserves, and therefore effort concentration continues even after the locally resident population becomes overexploited and collapses (Figs. 7b, d and e). In the worst of cases, this produces serial collapse of the areas surrounding reserves and eventually the collapse of the entire population.

In the absence of fishery squeeze, patterns of persistence and harvest are qualitatively similar for larval dispersal and adult movement. At small fractions of habitat in reserves (bottom half of Figures 5a–d), harvests are relatively insensitive to reserve width so long as reserves are of sufficient size to ensure persistence [46] and harvest increases with fraction of habitat in reserves. As noted by Moffitt *et al.* [25], harvests for a given small fraction of total habitat in reserves are considerably greater for the larval dispersal case than the adult movement case. Nevertheless, for both cases maximum harvests occur when the fraction of habitat in reserves is sufficient to ensure persistence for all reserve widths (i.e., network persistence occurs, top half of Figures 5a–d). In this case, maximum harvests occur for a network of many small reserves that cover just enough habitat to produce network persistence (i.e., along vertical axes of Figures 5a–d just above the area of non-persistence) [49], [50]. Maximum harvests are higher for the adult movement case, though differences are slight and most likely driven by the particulars of the functional relationship between harvest rate and harvest-per-recruit (Figure 1b). More importantly, high harvests are produced for a larger set of reserve configurations for adult movement than larval dispersal, though at greater overall fraction of habitat in reserves.

When fishery squeeze is included (Figures 5e–h and 6a–b), both persistence and harvest are qualitatively different for the adult movement than for larval dispersal. For the adult movement case, persistence requires reserves at least as large as the home range, and maximum harvests are lower than in the absence of fishery squeeze (e.g., ∼30–40% for the long-lived species and ∼35–45% for the spawn-first species). Furthermore, maximum harvests for the adult movement case occur at fractions in reserves approaching one and for reserves widths several times the home-range size (e.g., ∼4–7 times for all species). Le Quesne and Codling [29] also found maximum harvests require large reserve fractions, though their maximal harvests were higher for adult movement than larval dispersal. This discrepancy is due principally to their use of lower pre-reserve harvest rates, though a precise comparison is difficult due to differences in model formulation.

### Persistence with both adult movement and larval dispersal

As larval dispersal and adult movement often occur together, we examine their combined effects by comparing populations with varying levels of both processes. For simplicity, we consider only uniform effort distribution outside reserves. Populations are characterized by a total movement spatial scale given by the larval dispersal distance plus the adult home-range size. The adult home-range represents different fractions of this total movement scale, ranging from no adult movement (fraction of zero) to all adult movement (fraction of one).

**Figure 8:**
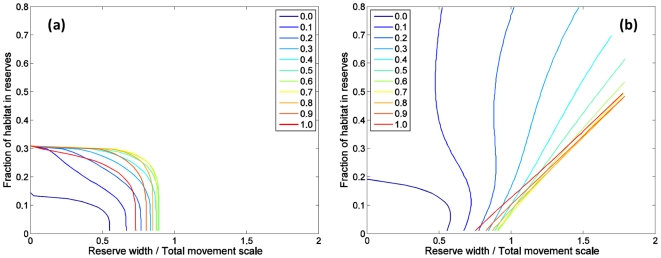
Border between persistence and collapse as a function of reserve width (in units of the ‘total movement scale’) and the fraction of habitat in reserves for the long-lived species. The ‘total movement scale’ is the sum of the larval dispersal distance and the adult home-range size. Blue to red curves are for different fractions of the total movement scale in adult home-range movement, ranging from larval dispersal only (0) to adult home-range movement only (1). In all cases, harvest effort is uniform outside reserves. In (**a**), there is no harvest effort redistribution after reserve creation, whereas in (**b**) total harvest effort is conserved before and after reserve implementation. Per recruit egg production is 25% of its unfished value in harvested areas.

Persistence occurs for fewer reserve configurations when larval dispersal and adult movement are combined (but each having a smaller spatial scale) than for exclusively one or the other process (Figures 8), rather than being intermediate between results for larval and adult cases, as might have been expected. At small reserve sizes (towards the left of Figures 8), the closure fraction necessary for persistence is the same for all cases but that of no adult movement. In this limit, any home-range size other than zero is always greater than the reserve size and persistence is driven by adult movement (Equation 15) irrespective of the amount of larval dispersal. This explains the rapid decrease in ‘network persistence’ when adults movement is added to a population with larval dispersal noted by Moffitt *et al.* [25]. For a single isolated reserve (horizontal axes in Figures 8), persistence requires larger reserves for most mixtures of adult and larval movement than for adult movement alone, except for rather small fractions in adult movement (<20% of the total movement scale).

## Discussion

Our results indicate that persistence of a population whose adults move within a home range requires significantly more area in reserves and/or larger reserves than for an equivalent population with larvae dispersing over the same spatial scale. Results are more pronounced for species beginning reproduction before first harvest (‘spawn-first’ species) since their reproductive capacity is more sensitive to harvest rate, though differences are relatively slight over the range of growth and reproduction configurations examined. The differences between adult movement and larval dispersal are accentuated when harvester movement is taken into account, producing patterns of persistence and harvest that are qualitatively different for the two movement processes. For example, even if harvest effort formerly in reserve areas is redistributed into non-reserve areas (i.e., ‘fishery squeeze’) and harvest effort concentrates spatially in response to increased prey densities near reserve edges (i.e., ‘fishing-the-line’), persistence of sedentary populations with dispersing larvae can always be achieved by creating either a single large reserve or placing more than a critical fraction of habitat in reserves (the latter being referred to as ‘network persistence’) (Figure 5g). For populations with mobile adults, persistence cannot be achieved solely by increasing the percentage in reserves, but rather requires individual reserve size be several times the adult home-range (e.g., >2 times for 50% in reserves in Figure 5h, for the long-lived species). Furthermore, though maximum harvests are roughly equivalent for the two movement types without fishery squeeze, they are considerably lower (e.g., ∼30–45% for the three species we considered) for populations with mobile adults when harvest effort redistribution is included, and require large fractions of habitat in reserves, producing extreme levels of harvest effort concentration. As harvester movement to areas of higher expected harvest and ‘fishery squeeze’ are likely to occur in the real world, our results highlight that ignoring harvester movement when it actually occurs can lead to dangerous overestimation of persistence in reserve networks.

The underlying cause of these differences in persistence for larval dispersal versus adult movement is more subtle than it might appear. One could assume that it is due to the fact that adult movement operates over the entire lifespan of an individual, whereas larval dispersal generally represents a small fraction of the lifespan. However, larvae dispersing outside of reserves are subject to harvest their entire lifetime, potentially having a greater negative effect on persistence. Which process is more detrimental is fundamentally linked to the results in Equations (10) and (12). Larval dispersal has the effect of averaging over egg production inside and outside reserves, whereas adult movement averages over harvest rate. As the relationship between harvest rate and reproductive capacity is convex, averaging over harvest rate is more detrimental. In biological terms, this is saying that persistence is better if some fraction of individuals are protected over their entire lifespan than if all individuals are protected a fraction of the time. As such, the result that adult movement is more detrimental for persistence than larval dispersal is general to all age-structured populations. Changes to model assumptions (such as, e.g., the type of density-dependent recruitment) are unlikely to alter this overall trend.

The results presented here include two aspects that appear at first glance paradoxical. The first is that fishery squeeze combined with adult mobility produces scenarios where no network of small reserves, no matter how dense, will lead to persistence and increasing the density of reserves can lead to collapse of networks that would have been persistent if the effort that was in closed areas had disappeared at the time of reserve creation. Effort redistribution, which will likely occur in the absence of effort restrictions or low harvester mobility, increases the harvest rate outside reserves as the fraction in reserves increases. With adult movement, as the fraction in reserves increases, fish spend more time inside protected areas but are also more likely to be harvested outside reserves due to increased fishing pressure. This leads to a net increase in effective harvest rate, even inside reserves, impeding persistence for networks of small reserves and eventually collapsing networks of larger reserves. This also explains the low maximum harvests for mobile adults with fishery squeeze because persistence is achieved by creating reserves of sufficient size that some individuals are inaccessible to harvest. These results highlight once more the need to effectively control harvest effort in non-protected areas for reserve implementation to be successful [8], [27], [51].

The second paradoxical result is that when both types of movement are present in the same population, persistence results are often worse than those for a population possessing just one of the two processes, even if the ‘total movement scale’ (the sum of larval dispersal distance and adult home-range size) is the same (Figures 8). The likely explanation for this is that larval dispersal reduces self-recruitment needed for persistence inside reserves at the same time that adult movement reduces the lifetime reproductive capacity of individuals recruiting to reserves. For populations whose larvae are indirectly dispersed through adult movement, such as some live-bearing sharks or species that do not separate feeding and reproductive habitats, these two movement processes are inevitably coupled and persistence will be negatively impacted.

These results have important consequences for spatial conservation efforts targeting mobile species. Larval dispersal has been a major focus of marine-reserve research, with significant effort being devoted to estimating larval dispersal scales [21], [52], [53], whereas adult movement has received less attention because many coastal species are sedentary and it is felt that long-distance larval dispersal is the dominant process affecting marine reserves. While the attention devoted to larval dispersal is by no means misplaced, the results here suggest that adult movement cannot be ignored in many cases. Home-range sizes of order 1–10 km cited for many California rockfish species [26], [35], for example, may be significant in terms of their effects on persistence for reserves that are often the same order of magnitude in size [25], particularly when the distribution and amount of harvest effort is not controlled.

Furthermore, conservationists and researchers have recently proposed using reserves for managing highly-mobile pelagic (e.g., tunas) and demersal (e.g., hakes) species [18]–[20]. These species undertake complex nomadic and migratory movements over hundreds to thousands of kilometers on monthly timescales [38], [54], [55]. Proposed solutions to creating effective reserve networks for these species include static or dynamic reserves that target certain sectors of spatially-structured populations (e.g., juveniles or spawners) [18], [22]. Though we have by no means examined the rather complex set of spatial migrations that may produce the spatial structure necessary for such ‘targeted’ approaches and marine reserve models indicate significant sensitivity of results to the precise temporal and spatial nature of movements [22], [29], [56], it is reasonable to assume that these results set a fairly high bar for the effective use of such approaches. Even relatively limited movement of individuals outside of pelagic reserves may significantly decrease reserve effectiveness, particularly if harvesters specifically target spillover (Figures 5d and h and 6a–b).

Despite these results, there is some evidence that marine reserves benefit mobile species [57]–[59]. These positive results have often been sources of new insights regarding the behavior of marine organisms and the interaction between behavior and conservation. For example, if habitat regeneration occurs inside reserves (e.g., through increased prey density), then residency time inside reserves may increase, thereby improving the value of reserves for mobile species. There are at least two cases where this is thought to have occurred involving emperor penguins in South Africa [58] and snappers in New Zealand [60]. Furthermore, it is now recognized that many mobile marine species possess specific subpopulations that are relatively sedentary (referred to as ‘behavioral polymorphism’) [22], [61]. Our results indicate that reserves may only protect these sedentary subpopulations, raising the possibility of strong selection for sedentarism [62]. It is our hope that the results presented in this paper will serve as a baseline for predicting responses of mobile species to reserve implementation and identifying when non-trivial species behaviors alter these predictions.

## Supporting Information

Appendix S1 Length-at-age relationships for yellowfin (*Thunnus albacares*) and skipjack tuna (*Katsuwonus pelamis*) populations of the Atlantic Ocean.
(DOC)

Appendix S2 Proof that per recruit egg production curves are always decreasing and convex.
(DOC)

Appendix S3 Border between persistence and collapse when harvest effort is uniformly distributed outside reserves.
(DOC)
