# Supplementary material for: Relative Impacts of Adult Movement, Larval Dispersal and Harvester Movement on the Effectiveness of Reserve Networks
Source: PLoS One. 2011 May 17;6(5):e19960. doi: 10.1371/journal.pone.0019960 (PMC3096657; doi:10.1371/journal.pone.0019960)
Supplement: Appendix S1 — Length-at-age relationships for yellowfin (Thunnus albacares) and skipjack tuna (Katsuwonus pelamis) populations of the Atlantic Ocean. (DOC) [file pone.0019960.s001.doc]

***Appendix S1.*** Length-at-age relationships for yellowfin (*Thunnus albacares*) and skipjack tuna (*Katsuwonus pelamis*) populations of the Atlantic Ocean.

Gascuel *et al.* [1] established for yellowfin tuna population of the Atlantic Ocean that length (*L*) can be predicted from the relationship:

|  | $L(A)= 37.8+8.93A+\left(137-8.93A\right)\left(1-e^{-0.808A} \right)^{7.49}$ | (S1.1) |
| --- | --- | --- |

where *A* is the age in years.

Hallier and Gaertner [2] established the following length-at-age relationship for skipjack tuna population of the Atlantic Ocean:

|  | $L(A)= 97.258\left(1-e^{-0.251A} \right)$ | (S1.2) |
| --- | --- | --- |

**References**

1. Gascuel D, Fonteneau A, Capisano C (1992) Modélisation d'une croissance en deux stances chez l'albacore (*Thunnus albacares*) de l'Atlantique est. Aquat Living Resour 5: 155–172.

1. Hallier JP, Gaertner D (2006) Estimated growth rate of the skipjack tuna (*Katsuwonus pelamis*) from tagging surveys conducted in the Senegalese area (1996-1999) within a meta-analysis framework.ICCAT-SCRS/2005/052
