# Supplementary material for: Relative Impacts of Adult Movement, Larval Dispersal and Harvester Movement on the Effectiveness of Reserve Networks
Source: PLoS One. 2011 May 17;6(5):e19960. doi: 10.1371/journal.pone.0019960 (PMC3096657; doi:10.1371/journal.pone.0019960)
Supplement: Appendix S2 — Proof that per recruit egg production curves are always decreasing and convex. (DOC) [file pone.0019960.s002.doc]

***Appendix S2.*** Proof that per recruit egg production curves are always decreasing and convex.

It will be assumed for simplicity that harvest mortality rate *f* and natural mortality rate *m* are constant with age, though this assumption is not essential to the argument. The per recruit egg production function *b* is given by:

|  | $b\left(f\right)= \int_{A0}^{\infty} e^{-\left(m+f\right)A}w\left(A\right)dA$ | (S2.1) |
| --- | --- | --- |

where *w*(*A*) *dA* is the (expected) egg production of a recruit between ages *A* and *A + dA*. Differentiating with respect to *f* yields:

|  | $b'\left(f\right)= -\int_{A0}^{\infty} Ae^{-\left(m+f\right)A}w\left(A\right)dA$ | (S2.2) |
| --- | --- | --- |

and

|  | $b''\left(f\right)= \int_{A0}^{\infty} A^{2}e^{-\left(m+f\right)A}w\left(A\right)dA$ | (S2.3) |
| --- | --- | --- |

Since the integrands are always positive, $b'\left(f\right)<0$ and $b''\left(f\right)\text{>0}$, i.e., *b* is decreasing and convex.
