# Supplementary material for: Relative Impacts of Adult Movement, Larval Dispersal and Harvester Movement on the Effectiveness of Reserve Networks
Source: PLoS One. 2011 May 17;6(5):e19960. doi: 10.1371/journal.pone.0019960 (PMC3096657; doi:10.1371/journal.pone.0019960)
Supplement: Appendix S3 — Border between persistence and collapse when harvest effort is uniformly distributed outside reserves. (DOC) [file pone.0019960.s003.doc]

***Appendix S3.*** Border between persistence and collapse when harvest effort is uniformly distributed outside reserves.

Previous studies [1,2] demonstrated that, for a population with sedentary adults and larvae dispersing over finite distances, the border between persistence and collapse is:

|  | $0=\left(\frac{1}{c^{2}}-c^{2}\right)\sinh\left(\frac{G}{c}\frac{\left(s-w\right)}{a}\right)\sin\left(Gc\frac{w}{a}\right)+2\left[\cosh\left(\frac{G}{c}\frac{\left(s-w\right)}{a}\right)\cos\left(Gc\frac{w}{a}\right)-1\right]$ | (S3.1) |
| --- | --- | --- |

with:

|  | $c=\left(\frac{b\left(0\right)-b\left(f_{c}\right)}{b\left(f_{c}\right)-b\left(f\right)}\right)^{\frac{1}{4}}$ | (S3.2) |
| --- | --- | --- |

and

|  | $G=\left[\frac{\left(b\left(0\right)-b\left(f_{c}\right)\right)\left(b\left(f_{c}\right)-b\left(f\right)\right)}{b \left(f_{c}\right)^{2}}\right]^{\frac{1}{4}}$ | (S3.3) |
| --- | --- | --- |

where *b* is the per recruit egg production *f* the instantaneous harvest rate, *f_c_* the harvest rate that reduces per recruit egg production to 35% of its unfished value in the absence of reserves, *m* the natural mortality rate, *a* the mean larval dispersal distance, *s* the spacing between reserves and *w* the width of a reserve.

In the limit of a single isolated reserve (i.e., in the limit $s\to\infty$) when the width of a reserve and the mean larval dispersal distance remain finite, the border between persistence and collapse in the larval dispersal case becomes:

|  | $0=\left(\frac{1}{c^{2}}-c^{2}\right)\sin\left(Gc\frac{w}{a}\right)+2\cos\left(Gc\frac{w}{a}\right)$ | (S3.4) |
| --- | --- | --- |

In this limit, the width of a reserve over the mean larval dispersal distance can be expressed as:

|  | $\frac{w}{a}=\frac{1}{Gc}\tan^{-1}\left(\frac{2c^{2}}{c^{4}-1}\right)$ | (S3.5) |
| --- | --- | --- |

In the case of mobile adults and non-dispersing larvae, when adult home range is finite, there is population collapse when collapse occurs in all the locations, i.e., when $f_{eff}\geq f_{c}$ in all locations (*f_eff_* is the effective harvest mortality rate experienced by mobile adult individuals). In other words, persistence is guaranteed when there is at least one location where $f_{eff}<f_{c}$. As reserves centers are the locations of the system where persistence is most likely, whether the population of interest will ultimately be persistent can be determined by evaluating if
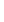
there, i.e.,
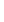
. Therefore, the border between persistence and collapse in the adult movement case when home range is finite is:

|  | $\left[\left[1\right]\right]=\nu\left(\frac{w}{2}\right)f-f_{c}=0$ | (S3.6) |
| --- | --- | --- |

It then comes that:

|  | $\begin{matrix} \left[\left[1\right]\right]=f\int_{\Omega_{F}} D_{A}(y,\frac{w}{2})dy-f_{c} \\ =\frac{f}{2a}\int_{-\infty}^{+\infty} e^{-\left\vert y-\frac{w}{2}\right\vert/a}dy-f_{c} \\ =\frac{f}{2a}\sum_{-\infty}^{+\infty} \int_{ks+w}^{ks+s} e^{-\left\vert y-\frac{w}{2}\right\vert/a}dy-f_{c}\forall k\in Z \end{matrix}$ | (S3.7) |
| --- | --- | --- |

where *a* is here the mean distance travelled from home range center.

After making a change of coordinate from$y$ to$y-\frac{w}{2}$, $\left[\left[1\right]\right]$ becomes:

|  | $\left[\left[1\right]\right]=\frac{f}{2a}\sum_{-\infty}^{+\infty} \int_{ks+\frac{w}{2}}^{ks+s-\frac{w}{2}} e^{-\left\vert y\right\vert/a}dy-f_{c}\forall k\in Z$ | (S3.8) |
| --- | --- | --- |

Splitting the sum into integrals over positive and negative values and switching $y$with $-y$ in the negative integrals, $\left[\left[1\right]\right]$ becomes:

|  | $\left[\left[1\right]\right]=\frac{f}{2a}\left[\sum_{1}^{\infty} \int_{ks+\frac{w}{2}}^{ks+s-\frac{w}{2}} e^{-y/a}dy+\sum_{0}^{\infty} \int_{ks+\frac{w}{2}}^{ks+s-\frac{w}{2}} e^{-y/a}dy\right]-f_{c}\forall k\in Z$ | (S3.9) |
| --- | --- | --- |

The two integrals are readily evaluated:

|  | $\left[\left[1\right]\right]=\frac{f}{2}\left[e^{-\frac{w}{2a}}-e^{-\frac{\left(s-\frac{w}{2}\right)}{a}}\right]\sum_{0}^{\infty} e^{-\frac{\left(k+1\right)s}{a}}+\frac{f}{2}\left[e^{-\frac{w}{2a}}-e^{-\frac{\left(s-\frac{w}{2}\right)}{a}}\right]\sum_{0}^{\infty} e^{-\frac{ks}{a}}-f_{c}\forall k\in Z$ | (S3.10) |
| --- | --- | --- |

Using the identity$\sum_{0}^{\infty} x^{\begin{matrix} \\ \alpha\end{matrix}}=\left(1-x\right)^{-1}\forall0<x<1$, it finally comes that the border between persistence and collapse in the adult movement case when home range is finite is:

|  | $0=f\frac{e^{-\frac{w}{2a}}\left(1-e^{-\frac{\left(s-w\right)}{a}}\right)}{1-e^{-\frac{s}{a}}}-f_{c}$ | (S3.11) |
| --- | --- | --- |

In the limit of a single isolated reserve (i.e., in the limit $s\to\infty$) when the width of a reserve and the mean distance travelled from home range center remain finite, the border between persistence and collapse in the adult movement case becomes:

|  | $0=fe^{-\frac{w}{2a}}-f_{c}$ | (S3.12) |
| --- | --- | --- |

In this limit, the width of a reserve over the mean distance travelled from home range center can be expressed as:

|  | $\frac{w}{a}=2\log\left(\frac{f}{f_{c}}\right)$ | (S3.13) |
| --- | --- | --- |

**References**

1. Kaplan DM, Botsford LW, Jorgensen S (2006) Dispersal per recruit: an efficient method for assessing sustainability in marine reserve networks. Ecol Appl 16: 2248-2263.

2. Van Kirk RW, Lewis MA (1997) Integrodifference models for persistence in fragmented habitats. B Math Biol 59: 107–137.
